# Clinical, Radiological, and Laboratory Features of Spinal Cord Involvement in Primary Sjögren’s Syndrome

**DOI:** 10.3390/jcm9051482

**Published:** 2020-05-14

**Authors:** Michaela Butryn, Jens Neumann, Leoni Rolfes, Claudius Bartels, Mike P. Wattjes, Nima Mahmoudi, Tabea Seeliger, Franz F. Konen, Thea Thiele, Torsten Witte, Sven G. Meuth, Thomas Skripuletz, Marc Pawlitzki

**Affiliations:** 1 Department of Neurology, Otto-von-Guericke University, 39120 Magdeburg, Germany; michaela.butryn@med.ovgu.de (M.B.); jens.neumann@med.ovgu.de (J.N.); claudius.bartels@med.ovgu.de (C.B.); 2 Department of Neurology with Institute of Translational Neurology, University Hospital Münster, 41849 Münster, Germany; leoni.rolfes@ukmuenster.de (L.R.); sven.meuth@ukmuenster.de (S.G.M.); 3 Department of Diagnostic and Interventional Neuroradiology, Hannover Medical School, 30625 Hannover, Germany; wattjes.mike@mh-hannover.de (M.P.W.); Mahmoudi.Nima@mh-hannover.de (N.M.); 4 Department of Neurology, Hannover Medical School, 30625 Hannover, Germany; seeliger.tabea@mh-hannover.de (T.S.); konen.felix@mh-hannover.de (F.F.K.); 5 Department of Clinical Immunology and Rheumatology, Hannover Medical School, 30625 Hannover, Germany; Thiele.Thea@mh-hannover.de (T.T.); Witte.Torsten@mh-hannover.de (T.W.)

**Keywords:** Sjögren’s syndrome, Spinal cord, Neurofilament light chain, Antibodies, Cerebrospinal fluid, Myelitis

## Abstract

Objective: To identify radiological and laboratory hallmarks in patients with primary Sjögren’s syndrome (pSS) presenting with spinal cord involvement. Methods: Clinical and laboratory routine parameters were analyzed in a retrospective multicenter case series of four patients who developed myelitis associated with pSS. Serological and cerebrospinal fluid (CSF) measurements of pSS associated anti-SSA(Ro)-antibodies were initiated, and CSF neurofilament light chain (NFL) levels were assessed. NFL values were compared with results from 15 sex- and age-matched healthy controls. Radiological assessment was performed using multi-sequence spinal cord magnetic resonance imaging. Results: Three of the four patients initially developed neurological signs suggestive of myelitis and were subsequently diagnosed with pSS. All patients presented a longitudinal spinal T2-hyperintense lesion in the cervical spinal cord, whereas only two patients showed pleocytosis and oligoclonal bands in the CSF. Median (range) CSF-NFL levels were significantly elevated in patients compared to controls (6672 pg/mL (621–50,000) vs. 585 pg/mL (357–729), *p* = 0.009). One patient showed sustained, highly increased NFL levels (50,000 pg/mL) in the initial assessment when radiological signs of axonal injury were still absent. Anti-SSA(Ro)-antibodies were found in the serum of three patients, while two patients additionally presented intrathecal anti-SSA(Ro)-antibody production. Elevated CSF-NFL levels and intrathecal synthesis of anti-SSA(Ro)-antibodies were associated with a relapsing and treatment-resistant disease course. Conclusion: Inflammatory spinal cord lesions associated with pSS are a rare but serious disease leading to severe disability. NFL and anti-SSA(Ro)-antibodies in CSF might serve as prognostic biomarkers and should be routinely assessed in patients with pSS.

## 1. Introduction

Primary Sjögren’s syndrome (pSS) is a chronic autoimmune disease characterized by lymphocytic infiltrations in secretory glands and extraglandular neurological manifestations [1,2]. The affection of the peripheral nervous system is more common than central nervous system (CNS) involvement. In particular, inflammatory spinal cord lesions are rarely present and have proven to be difficult to diagnose and treat [3,4,5].

Anti-SSA(Ro)-antibodies can be found in approximately 50% of patients suffering from pSS. These antibodies have a high diagnostic value and seem to be associated with extraglandular disease manifestations, although seronegative patients with CNS involvement have been described [6,7,8,9]. Interestingly, a direct pathogenic effect has been proposed, as anti-SSA(Ro)-antibodies can be detected serologically in patients with recurrent myelitis and without previous diagnosis of pSS [10].

The levels of neurofilament light chain (NFL) in serum or cerebrospinal fluid (CSF) are thought to reflect neuroaxonal damage and are proposed to have a predictive value concerning outcomes in patients with inflammatory and autoimmune diseases associated with myelitis [11,12].

Thus, we aimed to identify clinical, radiological, and laboratory hallmarks, including new potential biomarkers, in a detailed case series of patients with pSS presenting with spinal cord involvement.

## 2. Material and Methods

### 2.1. Patients and Biomaterials

Patients were recruited at the Departments of Neurology of the University Hospitals Magdeburg, Münster, and Hannover, all located in Germany. CSF and serum samples from 15 sex- and age-matched healthy controls recruited in Magdeburg were included for comparison. All controls reported non-specific complaints and underwent lumbar puncture (LP) during a routine diagnostic examination conducted to rule out any neurological condition. None of the controls was diagnosed with a specific neurological disorder or showed any specific abnormalities during the neurological examination. In addition to the clinical classification, all controls fulfilled the following laboratory criteria defining a normal CSF: < 5 cells/μL, < 2.0 mmol/L lactate, no disruption of the blood-CSF-barrier function (defined by the albumin CSF/serum quotient), no oligoclonal bands (OCB), and no intrathecal synthesis of the immunoglobulins (Ig) IgG, IgA, and IgM [13]. In addition, we calculated the antibody specificity indices (ASI) for each case to detect intrathecal anti-SSA(Ro) antibody synthesis. The index is based on the Serum/CSF-quotient of specific antibodies and hereby quantifies their production in the serum vs. in the CSF. It is, therefore, designed to differentiate between passive antibody transmission from serum to CSF (index ≤ 1.5) and intrathecal antibody production (index > 1.5).

### 2.2. NFL and Anti-SSA(Ro)-Antibody Analyses

For all patients, a LP was performed at each respective study site according to standard protocols. The LP for all controls was conducted in Magdeburg. At each study site, CSF cells were counted manually using a Fuchs-Rosenthal chamber. Concentrations of Albumin, IgG, IgM, and IgA in serum and CSF were measured by kinetic nephelometry. Intrathecal synthesis of IgG, IgA, and IgM was calculated according to Reiber’s revised hyperbolic function. Isoelectric focusing was applied to detect CSF specific oligoclonal bands. All samples for CSF-NFL and antibody measurements were stored at −80 °C and shipped on dry ice. CSF-NFL levels were determined in Magdeburg using commercially available ELISA kits (UmanDiagnostics NF-light®, Umea, Sweden, catalog number 10-7001 CE). Each measurement was performed together with a blank and a commercial positive and negative control provided by the manufacturer. Anti-SSA(Ro)-antibodies both in sera and CSF were measured in Hannover using EliA (ThermoScientific, Waltham, MA USA). Samples were measured in serial procedures and not in batches.

### 2.3. Magnetic Resonance Imaging (MRI) Protocols

Multi-sequence MRI (1.5 Tesla) of the spinal cord was performed in all patients and included a sagittal T2-weighted turbo-spin echo, a short tau inversion recovery sequence, and a T1-weighted postcontrast sequence covering the cervical and upper thoracic cord. Additionally, axial T2-weighted turbo-spin echo sequences were used. Furthermore, multi-sequence MRI of the head was performed in patients 1, 3, and 4, and included an axial T1- (pre- and postcontrast), a T2-weighted turbo-spin echo sequence and an axial diffusion-weighted imaging sequence. Moreover, a 3D sagittal T2-FLAIR-weighted sequence was applied in patients 1 and 4 and an axial T2-FLAIR-weighted sequence in patient 3.

### 2.4. Standard Protocol Approval, Registration, and Patient Consent

This study was performed according to the Declaration of Helsinki and approved by the local ethics committees (Magdeburg: No. 07/17, Münster: 2016-053-f-S, Hannover: No. 8270_BO_S_2019). All patients gave written informed consent.

## 3. Results

### 3.1. Cohort

Demographic, clinical, and laboratory findings are provided in Table 1. Four patients from Magdeburg (n = 1), Münster (n = 1), and Hannover (n = 2) with inflammatory spinal cord lesions associated with pSS were enrolled. Three of the four patients were female. The median age at baseline was 51 years (range 39–59 years). Patient 2 was diagnosed with pSS before any neurological symptoms occurred. The remaining three patients developed neurological signs of spinal cord involvement prior to diagnosis. The patients were classified according to the 2017 ACR-EULAR score (American College of Rheumatology/European League against Rheumatic Disease) for primary Sjögren’s syndrome and the EULAR Sjögren’s syndrome disease activity index (ESSDAI) at baseline [14,15,16]. Notably, none of the patients fulfilled the current criteria for neuromyelitis optica spectrum disorders. All patients tested negative for aquaporin-4- and myelin oligodendrocyte glycoprotein (MOG)-antibodies [17].

### 3.2. Case 1

**Figure 1:**
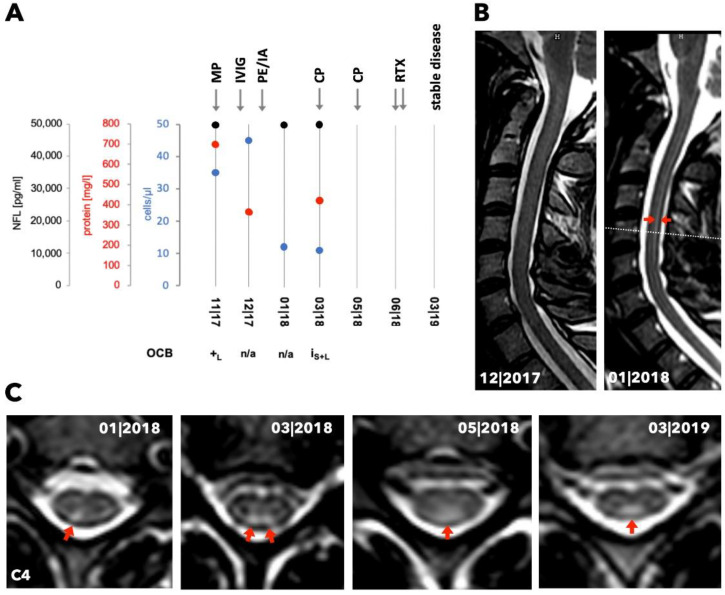
Longitudinal laboratory and radiological data and treatment changes for patient 1. (**A**) Chart shows the course of cell count, concentrations of protein and neurofilament light chain (NFL) as well as the occurrence of oligoclonal bands (OCB) in the CSF over time (n/a = not examined, +_L_ = detected in CSF, I_S+L_ = identical OCB in serum and CSF). Arrows indicate time points of treatment (MP = methylprednisolone, IVIG = intravenous immunoglobulin, PE/IA = plasma exchange/immunoadsorption, CP = cyclophosphamide, RTX = rituximab). (**B**) Panel displays MRI (T2 sagittal plane) at two different time points. The red arrows indicate the T2-enhancement of the posterior part of the spinal cord. The dotted line presents the height of the transversal planes shown in (**C**). (**C**) Transversal planes of the cervical spinal cord (C4) show T2-enhancement over time in the posterior columns, indicated by the red arrows.

A 39-year-old Caucasian female with known hypothyroidism (without evidence of a Hashimoto thyroiditis), alopecia of undeterminable cause, and sicca symptoms developed progressive sensory ataxia of the lower limbs and a mild paraparesis in 10/2017. MRI examinations of the brain and spinal cord were inconspicuous in 10/2017 and 12/2017. Lower limb somatosensory evoked potentials (SEP) showed axonal affection, whereas motor and sensory neurographies provided no evidence of neuropathy. Repeated CSF investigations revealed a pleocytosis (12–45 cells/µL) and OCB in CSF (Figure 1A). Immunological analysis of autoantibodies revealed elevated anti-SSA(Ro)-antibodies, while other serum markers for vasculitis or demyelinating CNS disorders were negative. Xerophthalmia and xerostomia were identified, and thus, pSS was diagnosed. Notably, the Schirmer test was not performed at that time (ACR-EULAR score of 3 points at baseline) but turned out positive a few months later. Intravenous (IV) treatment with methylprednisolone (1 g/day for 5 days) in 12/2017 did not lead to clinical improvement. Subsequently, IVIg (30 g/day for 3 days) was administered. Due to a lack of improvement, the patient received six courses of plasmapheresis/immunoadsorption (12/2017–01/2018), leading to temporary clinical stabilization. Three months after disease onset, spinal cord MRI showed a right-sided homogeneous T2-hyperintense lesion without T1-gadolinium enhancement in the posterior column of the cervical spinal cord, which had not been visible on previous MRI scans (Figure 1B,C). The initial MRI of the brain showed a small number of punctate deep white matter lesions suggestive of vascular origin without T1-gadolinium enhancement.

During rehabilitation, gait disturbance due to sensory ataxia worsened, leading to wheelchair dependency. Furthermore, the upper limbs were now affected, and the patient developed a bilateral hearing loss. Anti-inflammatory therapy was complemented by two cycles of IV cyclophosphamide (04/2018 and 05/2018, total dose: 900 mg/m^2^ body surface area). During therapy, an additional left-sided T2-hyperintense lesion, without T1-gadolinium enhancement, was detected in the posterior column of the cervical and thoracic spinal cord (Figure 1C). The right-sided posterior column T2-hyperintense lesion now extended into the thoracic cord. Consequently, two doses of 1000 mg rituximab (05/2018 and 06/2018) were applied. Disease progression could be stopped under ongoing rituximab treatment (every 6 months), but neurological recovery was not achieved.

### 3.3. Case 2

**Figure 2:**
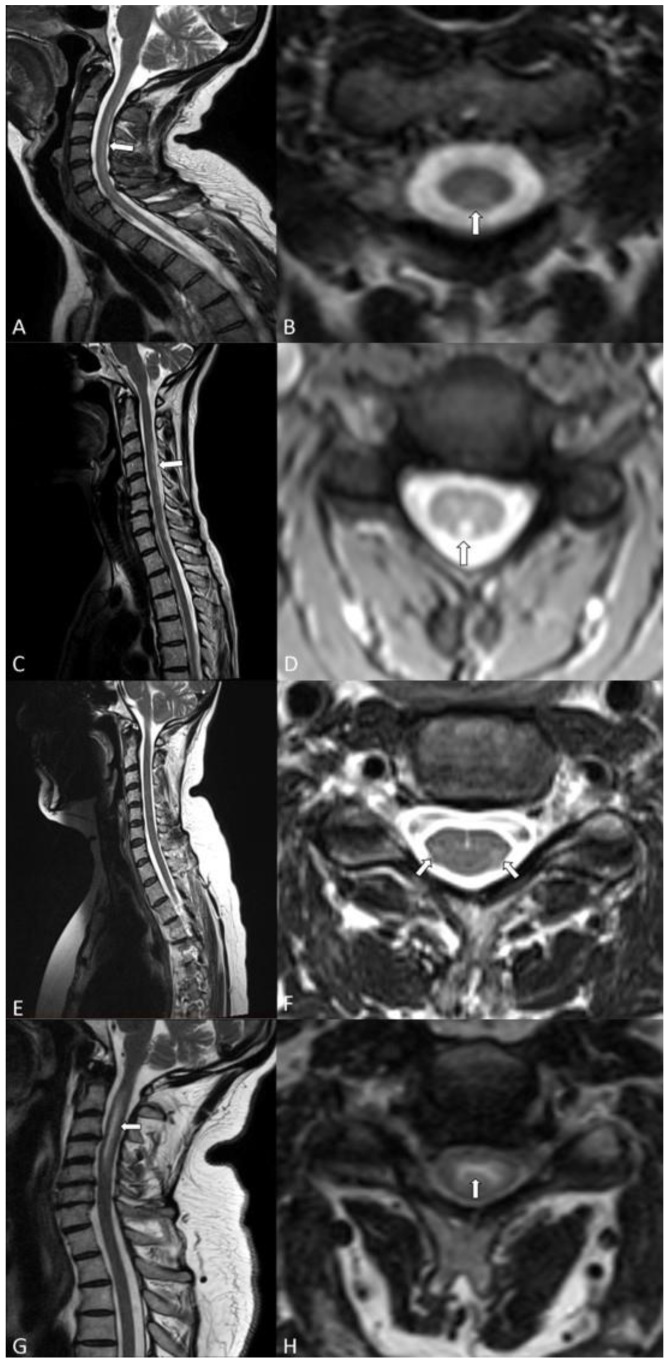
Cervical spinal cord MRI of all patients, with sagittal (left) and axial (right) T2-weighted sequences. (**A**,**B**) *Patient 1:* White arrow indicates a right-sided homogeneous T2-hyperintense lesion in the posterior columns. (**C**,**D**) *Patient 2:* White arrow indicates a T2-hyperintense longitudinally extensive spinal cord lesion in the posterior columns. (**E**,**F**) *Patient 3:* White arrows indicate a long segment T2 lesion of the lateral pyramidal tracts and lateral spinothalamic tracts. (**G**,**H**). *Patient 4:* White arrow indicates a long segment T2-hyperintense lesion mainly involving the central grey matter.

A 50-year-old Caucasian female with pSS, diagnosed in 2014, and evidence of elevated anti-SSA(Ro)-antibodies under treatment with hydroxychloroquine and intermittent use of oral glucocorticosteroids (GCS), developed a mild dysesthesia of the upper and lower limbs and slight sensory ataxia in 2016. Lower limb SEP, peripheral nerve conduction studies of upper and lower limbs, and CSF analysis were inconspicuous. The cervical MRI identified a T2-hyperintense longitudinally extensive spinal cord lesion without T1-gadolinium enhancement in the posterior column as the cause for neurological symptoms (Figure 2C,D). Disease stabilization was achieved with the additional administration of methotrexate on top of the established oral therapy with hydroxychloroquine and GCS.

### 3.4. Case 3

A 55-year-old Caucasian female with known primary biliary cholangitis developed a progressive dysesthesia of the upper and lower limbs and a mild tetraparesis in 2018. The spinal cord MRI showed a long segment T2 lesion without T1-gadolinium-enhancement in the lateral pyramidal tracts and lateral spinothalamic tracts of the cervical spine (Figure 2E,F). The head MRI displayed multiple punctate T2-hyperintense lesions in the deep and periventricular white matter and the dorsal pons. Some periventricular and deep white matter lesions showed a perivascular distribution pattern. None of the lesions indicated a disrupted blood-brain barrier.

Pathological changes in lower limb SEPs were recorded, whereas nerve conduction examinations of upper and lower limbs showed no impairment of motor and sensory nerves. CSF measurements did not reveal inflammatory signs. In line with the evidence of elevated anti-alpha-fodrin antibodies, objective xerophthalmia, and a pathological minor salivary gland histology, an inflammatory spinal cord lesion associated with pSS was diagnosed. Therapy was initiated with IV cyclophosphamide (cumulative dose 4750 mg/m^2^ body surface), oral GCS, and finally with rituximab (initially 2 × 1000 mg, hereafter administered every 6 months), under which disease progression could be stopped.

### 3.5. Case 4

A 52-year-old Caucasian male with known psoriasis developed a tetraparesis in 2018. The spinal cord MRI showed a long segment T2-hyperintense lesion with marginal T1-gadolinium-enhancement, mainly involving the central grey matter of the cervical cord (Figure 2G,H). The head MRI presented a small number of punctate deep white matter T2-lesions without T1-gadolinium-enhancement suggestive of vascular origin and a lacunar infarct in the left periventricular white matter. Due to the evidence of elevated anti-SSA(Ro)-antibodies and objective xerophthalmia and xerostomia, myelitis associated with pSS was diagnosed. Lower limb SEPs were conspicuous, and CSF measurements showed a pleocytosis and OCB. Treatment was initiated in 03/2018 with IV corticosteroids (5 × 1 g) and 5 cycles of immunoadsorption, followed by one course of IV rituximab (2 × 1000 mg). Because of disease progression, the administration of IV corticosteroids (5 × 1000 mg) was repeated without any clinical benefit. Thus, the treatment was escalated to another 5 courses of immunoadsorption and IV cyclophosphamide in 04/2018, under which the neurological symptoms stabilized. Due to relapse in 05/2018, therapy was again escalated with five courses of immunoadsorption and the second cycle of rituximab (1000 mg), resulting in an improvement of the tetraparesis. To achieve long-term clinical stabilization, IV cyclophosphamide was applied at a cumulative dose of 4500 mg/m^2^ body surface over the next months, and immunosuppressive treatment was hereafter continued with azathioprine while periodic courses of rituximab were administered additionally every 6 months. Disease progression could be stopped.

### 3.6. MRI Pattern

The radiologically examined morphology of the spinal cord revealed a punched-out affection of anatomically defined longitudinal tracts with a long segment involved in all four patients. Patients 1 and 2 presented T2-hyperintense lesions, mainly affecting the dorsal columns of the spinal cord (Figure 2A–D). Patient 3 showed T2-hyperintense lesions in the lateral pyramidal tracts and lateral spinothalamic tracts of the cervical spine on both sides (Figure 2E,F). While seeing primary white matter affection of the spinal cord in patients 1, 2, and 3, we detected an isolated long segment T2-hyperintense lesion mainly located in the central grey matter in patient 4 (Figure 2G,H).

### 3.7. Antibody Analyses

Patients 1, 2, and 4 presented elevated levels of anti-SSA(Ro)-antibodies in sera and CSF. An elevated antibody specificity index (ASI) was detectable in patients 1 (2.2) and 4 (5.1), while patient 2 showed no elevated ASI (1.0). We could not detect anti-SSA(Ro)-antibodies in the serum or CSF of patient 3.

### 3.8. NFL Analyses

Although CSF-NFL levels in patient 2 were normal [12], the median (range) CSF-NFL value of pSS patients was significantly higher compared to controls (6672 pg/mL (621–50,000) vs. 585 pg/mL (357–729), Mann-Whitney U test: p = 0.009). We initially detected sustained and highly increased NFL levels in patient 1 (permanently at 50,000 pg/mL), when imaging signs of axonal injury were still absent (Figure 1A).

## 4. Discussion

Due to the aggressive disease course involving the CNS, all patients were extensively assessed, including radiological, serological, and CSF analysis.

Previous reports have described a rare occurrence of spinal cord manifestations associated with pSS [10,19,20]. Typically, patients present with partly severe neurological symptoms and underlying early longitudinally extensive transverse myelitis as a correlating radiological feature [21]. However, the pattern of spinal cord affection has not yet been fully identified. Our findings regarding the distinct affection of longitudinal spinal tracts suggest a preferred pattern for myelitis associated with pSS. Further studies are needed to verify this MRI pattern. Nevertheless, the presence of such MRI findings should initiate diagnostic procedures of pSS, which include measurement of the tear and saliva flow, anti-SSA(Ro)-antibodies, and, if these are absent, a salivary gland biopsy. Considering the initially normal spinal cord MRI in patient 1 with subsequent spinal cord inflammation during the further disease course, and looking at the delicate structures involved in all our cases, underestimation and delayed diagnosis of this neurological manifestation in patients with pSS is obvious. Additionally, our case series emphasizes that the extent of the spinal cord lesion does not provide prognostic information about the neurological disability, while this association between the clinical state and the length of the spinal cord lesion has been described for other autoantibody-associated demyelinating CNS disorders [22,23].

To date, the relationship between anti-SSA(Ro)-antibodies and neurological manifestations in pSS is not sufficiently understood, in particular since both antibody-negative courses and isolated antibody-positive associated myelitis have been described [7,8,10,19,24,25]. Only a few study groups have investigated the impact of CSF-anti-SSA(Ro)-antibodies in patients with neurological involvement of primary rheumatological disorders such as pSS or systemic lupus erythematosus, reporting an intrathecal synthesis occurring partly prior to systemic manifestation [9,26]. Due to the severe clinical decline in our two patients with intrathecal synthesis of anti-SSA(Ro)-antibodies, we propose that CSF antibody measurement should be a standard assessment in patients with a suspected neurological manifestation of pSS.

CSF-NFL measurements have recently been discovered to possess biomarker qualities for different neurological disorders and can be applied to assess early axonal damage and reflect early disease activity [27], even in the absence of morphological findings [28]. Interestingly, in our case series, patients with highly elevated CSF-NFL levels showed the most severe clinical course, with multiple relapses and persistent neurological deficits despite aggressive immunosuppressive treatments, comparable to reports of other demyelinating CNS diseases [29,30,31,32]. Moreover, for patient 1, the latency between highly increased CSF-NFL levels and the first indicative MRI changes was remarkable [28]. Additionally, the intrathecal synthesis of anti-SSA(Ro)-antibodies was associated with high NFL levels, suggesting antibody-mediated axonal damage [10].

## 5. Conclusions

In conclusion, physicians need to keep in mind that in rare cases, imaging findings can be absent, particularly in the early disease course, or difficult to detect since fine longitudinal tracts can be involved in an isolated manner. However, absent radiological findings should not prevent from further diagnostic procedures necessary to identify a possible pSS. Moreover, our case series implies that CSF-NFL and intrathecal synthesis of anti-SSA(Ro)-antibodies are reliable and early markers for severe spinal cord involvement associated with pSS. We therefore strongly suggest that both values are routinely assessed to predict pSS-related CNS involvement even in the absence of radiological findings or systemic disease activity. Nevertheless, this study is limited because of its low number of analyzed patients, which is a common problem in rare diseases. Further investigations in larger cohorts are needed to verify the pattern of spinal cord affection and to clarify the association between the clinical course, NFL levels and the intrathecal production of anti-SSA(Ro)-antibodies.

## Figures and Tables

**Table 1:** Clinical, radiological, and laboratory findings.

	Patient 1	Patient 2	Patient 3	Patient 4
Study center	Magdeburg	Münster	Hannover	Hannover
Sex	female	female	female	male
Age (years)	39	50	55	52
Disease duration (months) of Sjögren’s syndrome	0	60	27	1
Leading neurological symptoms	severe sensory ataxia, mild paraparesis	slight sensory ataxia, dysesthesia	dysesthesia, mild sensorimotor tetraparesis	sensorimotor tetraparesis
Further organ manifestation	sicca symptoms, alopecia, hearing loss	sicca symptoms, arthralgias	sicca symptoms	sicca symptoms, arthralgias
ACR-EULAR-Score (baseline)	3	4	4	5
ESSDAI (baseline)	15	20	17	17
Histology (lip salivary gland, Chisholm & Mason grade [18])	0	n/a	4	1
MEP (tibialis anterior muscle)	abnormal	unremarkable	not done	unremarkable
SEP (tibial nerve)	abnormal	unremarkable	abnormal	abnormal
Motor/sensory neurography u/l limb	unremarkable	unremarkable	unremarkable	unremarkable
Brain T2 lesions	no	no	yes	yes
Extent spinal cord T2 lesion	C3-T5	C3-C6	C2-7	C1-6; T12
Gd^+^ of spinal cord lesion	no	no	no	yes
CSF cell count (n/µl)	45	0	3	39
CSF OCB	positive	negative	negative	positive
CSF TPC (mg/l)	355	380	394	462
Qalb	10.9	5.0	5.01	6.99
CSF NFL (pg/ml)	50,000	621	3161	9984
Serum SSA(Ro)- antibodies total (U/ml)	58	2080	0	184
CSF SSA(Ro)- antibodies total (U/ml)	0.75	5.3	0	6.1
ASI SSA(Ro)	2.2	1	/	5.1
Treatment history	IV GCS rituximab IV cyclophosphamide PE/IA IV immunoglobulins	oral GCS hydroxychloroquine methotrexate	oral GCS rituximab IV cyclophosphamide	IV + oral GCS rituximab IV cyclophosphamide IA azathioprine

ACR-EULAR-Score = American College of Rheumatology-European League against Rheumatic Disease-Score for primary Sjögren’s Syndrome, ASI = antibody specificity index, CSF = cerebrospinal fluid, ESSDAI = EULAR Sjögren Syndrome Disease Activity Index,, Gd^+^ = Gadolinium enhancement, GCS = glucocorticosteroids, IA = immunoadsorption, IV = intravenous, NFL = neurofilament light chain, OCB = oligoclonal bands, MEP = Magnetic evoked potentials, n/a: not examined, PE = Plasma exchange, Qalb = albumin quotient, SEP = Sensory evoked potentials, TPC = total protein count, u/l limb = upper/lower limb. ASI indicates the CSF/serum difference of antibody amounts per weight unit IgG (normal < 1.5).
